# Applying Nitrogen Site-Specifically Using Soil Electrical Conductivity Maps and Precision Agriculture Technology

**DOI:** 10.1100/tsw.2001.95

**Published:** 2001-10-17

**Authors:** E.D. Lund, M.C. Wolcott, G.P. Hanson

**Affiliations:** Veris Technologies, Salina, KS 67401, USA

**Keywords:** global positioning systems, GPS, geographic information systems, GIS, precision, site-specific, soil EC, electrical conductivity, variable rate, N, nitrogen, yield, soil sampling, cotton, corn, wheat, overapplication, soil texture, yield goal, productivity, rank growth, management zones

## Abstract

Soil texture varies significantly within many agricultural fields. The physical properties of soil, such as soil texture, have a direct effect on water holding capacity, cation exchange capacity, crop yield, production capability, and nitrogen (N) loss variations within a field. In short, mobile nutrients are used, lost, and stored differently as soil textures vary. A uniform application of N to varying soils results in a wide range of N availability to the crop. N applied in excess of crop usage results in a waste of the grower’s input expense, a potential negative effect on the environment, and in some crops a reduction of crop quality, yield, and harvestability. Inadequate N levels represent a lost opportunity for crop yield and profit. The global positioning system (GPS)-referenced mapping of bulk soil electrical conductivity (EC) has been shown to serve as an effective proxy for soil texture and other soil properties. Soils with a high clay content conduct more electricity than coarser textured soils, which results in higher EC values. This paper will describe the EC mapping process and provide case studies of site-specific N applications based on EC maps. Results of these case studies suggest that N can be managed site-specifically using a variety of management practices, including soil sampling, variable yield goals, and cropping history.

## INTRODUCTION

Determining the proper amount of nitrogen (N) to be applied to an agricultural field is a source of debate and discussion among growers, input suppliers, and researchers. Some commonly accepted methods for devising N rates for corn, wheat, and cotton production are (1) the establishment of a yield goal that determines the overall N level needed for the crop to achieve that goal[1,2], (2) soil sampling to determine the ability of the soil to supply a portion of the N needed by the crop[2,3], and (3) local university research of N response functions. In addition, most growers' decisions are also influenced by traditional fertilization practices in their area and by their own knowledge and experiences gained by working their land. In actuality, decisions on N rates are usually based on a combination of these methods.

A potentially serious environmental and economic problem with any single N rate is that there are typically areas within a field that need a different amount than the uniform rate. As shown in Fig. 1, available N as determined by soil sampling on a 0.3-ha grid can vary significantly within a 50-ha field. A uniform rate based on a single sample from the low-N areas would result in overapplication on the areas already containing high N levels. Also, crop yields can vary widely within a field, and frequently these exhibit spatially structured patterns, as shown in Fig. 2. Devising a single N rate based on one yield goal for this field would likely result in overapplication to areas that have shown over time to have lower yield potential.

With the advent of precision agriculture technologies, such as global positioning systems (GPS), geographic information systems (GIS), and soil electrical conductivity (EC) mapping,

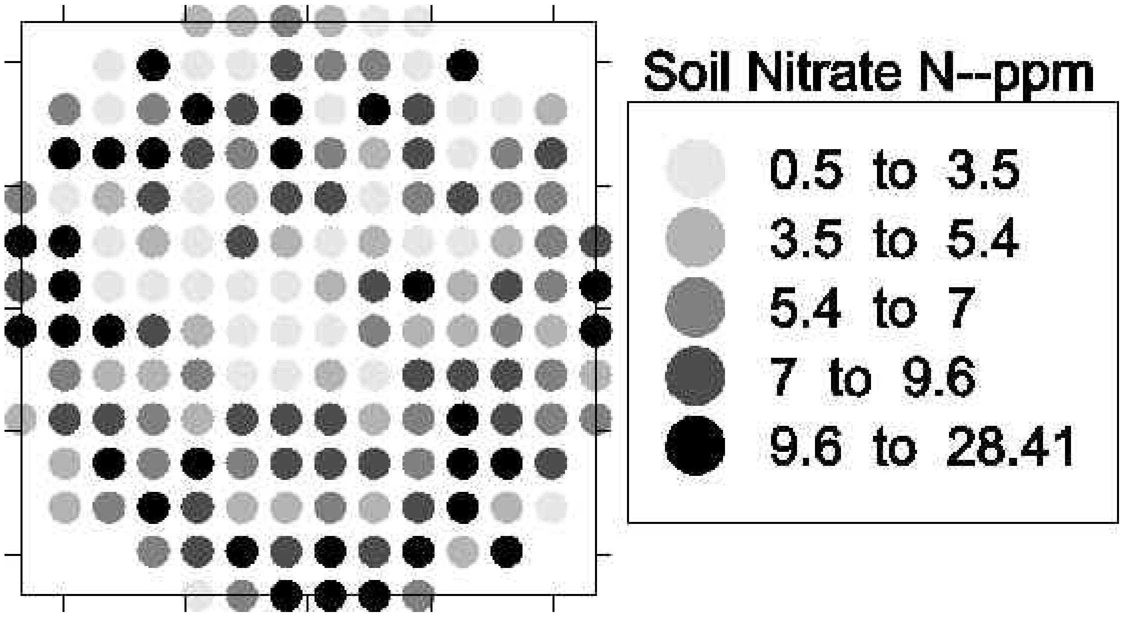

**FIGURE 1.** Variability of N levels within a 50-ha field (0 to 60 cm depth; fall sampling).

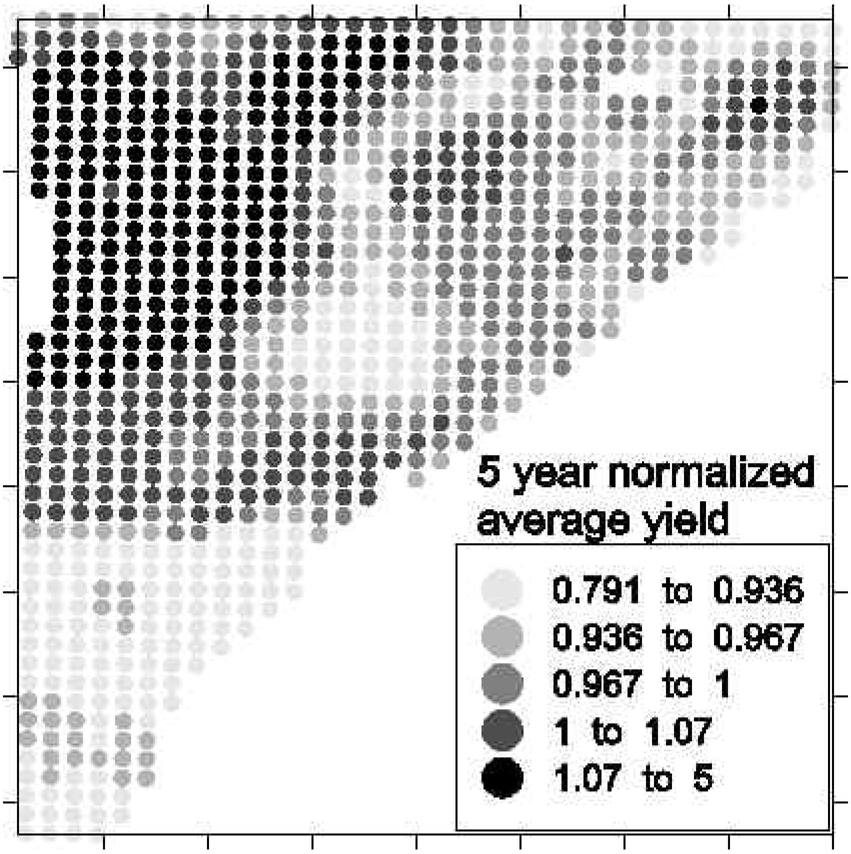

**FIGURE 2.** Variability of historical yield within a 10-ha field.

growers and their consultants can soil-sample and record geo-referenced field characteristics with 1- to 3-m accuracy. They are now beginning to apply N based on subfield criteria, and varying rates to more closely meet the site-specific demands of the crop. Specifically, these tools can be used in a site-specific manner to improve N efficiency by assisting in determining the availability of N in the soil and estimating the amount of N needed by the crop.

## MATERIALS AND METHODS

In order to determine the N needs of a field, two questions about the spatial variability of the field must be answered: where does the yield potential vary, and where does N availability vary? Each of the case studies presented here include the use of soil EC mapping as a layer of soils information used to help address these questions.

## Soil N Availability

The usefulness of soil EC in precision agriculture stems from the fact that it typically correlates well with soil texture: sands have a low conductivity, silts have a medium conductivity, and clays have a high conductivity[4], as shown in Fig. 3. In turn, the texture of soil strongly affects N activity in it and N movement through it[5,6].

The rapid mapping of a field’s soil EC can be accomplished by mobilized equipment like the Veris® 3100 Soil EC Mapping System. As the cart is pulled through the field, the system acquires conductivity measurements and geo-references them using a GPS receiver. When used on 15- to 20-m swaths at speeds of up to 12 km/hr, the system produces between 40 and 100 samples per hectare. Contact methods, like that used by the Veris® model 3100, use at least four electrodes that are in physical contact with the soil to inject a current and measure the voltage that results (Fig. 4).

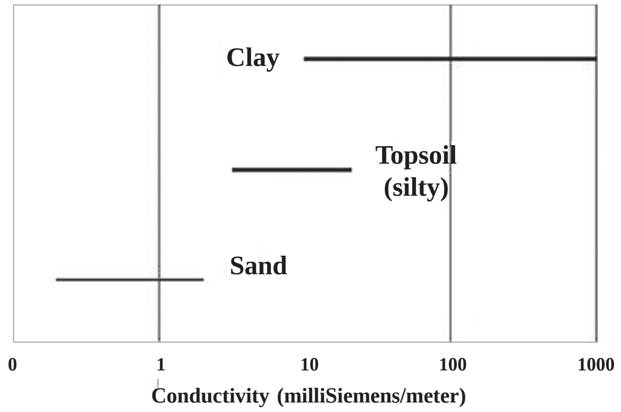

**FIGURE 3.** Soil EC depends on soil texture and other properties.

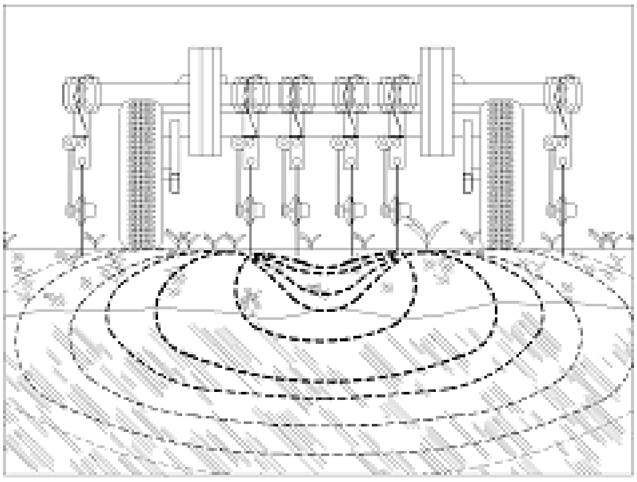

**FIGURE 4.** The Veris® 3100 soil conductivity mapping system employs two arrays to investigate soil at two depths, 0 to 25 cm and 0 to 75 cm.

Mapping the soil EC of a field can serve as an effective proxy for mapping the soil texture of the field, at a significantly reduced cost, as shown in Fig. 5. While it is not yet possible to predict actual N levels directly from soil EC, it is possible to use soil EC as a surrogate measurement for the properties that directly affect N levels[7]. Soil EC maps can be used effectively for guiding soil sample locations and in a GIS as a geo-referenced soils layer.

## N Needed for Crop Production

Soil EC measurements are closely correlated with soil texture and nutrient holding capacity[4,8], which are important factors in crop yield. EC has shown to relate closely to other soil properties that can influence productivity, including organic carbon content[7], cation exchange capacity (CEC)[8], soil depth[9], water holding capacity/drainage[10], and salinity[11]. Therefore, EC maps often correlate well with crop yield maps[12]. If low-yielding areas are stable across multiple years of various growing conditions, and if those same areas correlate with soil conditions that typically accompany lowered crop yields, a grower may elect to set a lower yield goal in these areas and reduce his N inputs accordingly (see Fig. 6).

## RESULTS

The following examples describe how crop producers in three different regions of the U.S. are practicing these innovations. In each of these case studies, the traditional whole-field approach

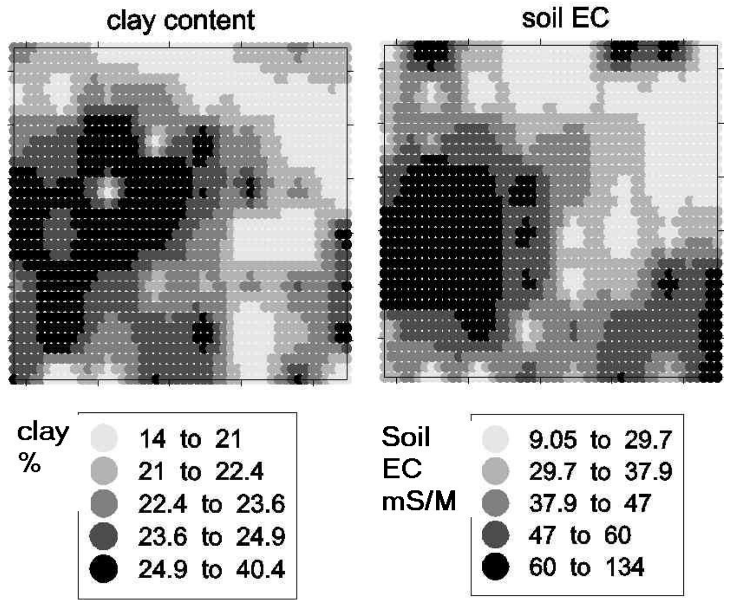

**FIGURE 5.** Clay content and soil conductivity from a 16-ha Iowa field.

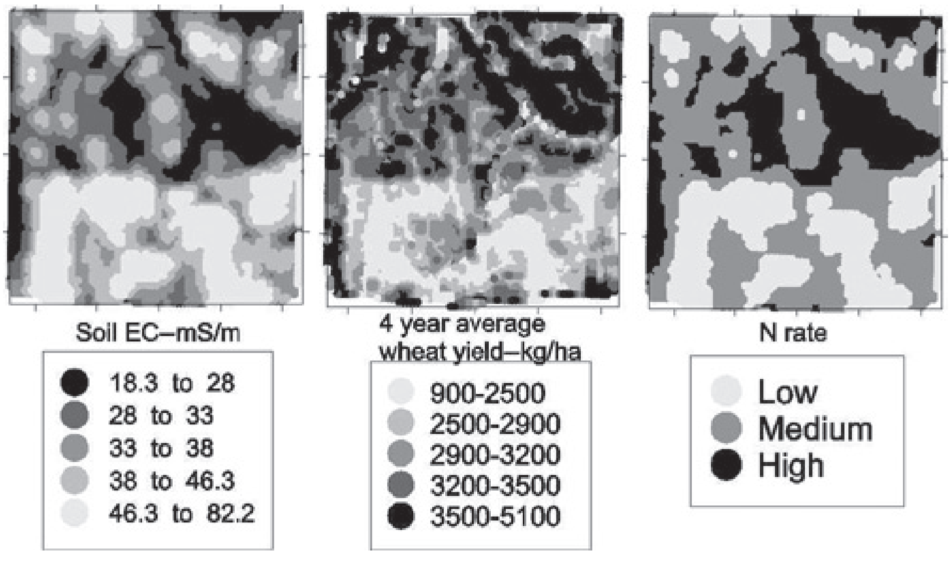

**FIGURE 6.** Maps of EC, historical crop yields, and a yield goal-based N rate from a 65-ha field.

to N management for that cropping system is fine-tuned using site-specific technology.

## Case Study No. 1

The first case study is from the nonirrigated, predominately cereal grain-producing area of the Dakotas. The customary practice in this area is to apply one N rate to an entire field, based on a single sample of soil cores composited from various areas of the field. These cores are typically collected from soil depths of 0 to 180 cm, and may be segmented at various levels within the sample profile. Research has shown that there is considerable within-field variability in the available N levels, and that this variability is related to the movement of water and nutrient movement in and through the landscape[3]. Research has also shown that the sampling density needed to properly capture this variability would require a 0.4-ha grid[13]. Due to the expense of the deep nitrate sampling and the relatively low value of the commodity crops being produced, soil sampling at this intensity is not practical. An alternative is to sample the field guided by layers of information that correlate to water and nutrient movement, such as topography and soil EC, and to nutrient usage, such as yield maps and remote crop imagery[3].

This approach has been commercialized by MZB Technologies of Jamestown, ND on more than 20,000 ha since 1998. Under the MZB system, soil EC, precise elevation data, crop vigor imagery, and other layers of data are collected, as in Fig. 7.

These data are clustered into zones of similarity using a GIS program. Soil sample locations are determined using the grower's input and the zone map (see Fig. 8).

The field is sampled, then the soil samples are composited within each zone and laboratory analyzed. Bar graphs are produced that show the different N levels in each zone (see Fig. 9). An N rate is set for each soil zone, taking into account the grower's input, soil sample results, and yield goals (see Fig. 10).

Growers have been encouraged to leave uniform rate test strips in their fields and use yield monitors to compare them with the results of variable rate N. Results from 33 sites in Brown County, South Dakota and in Stutsman, Lamoure, and Dickey

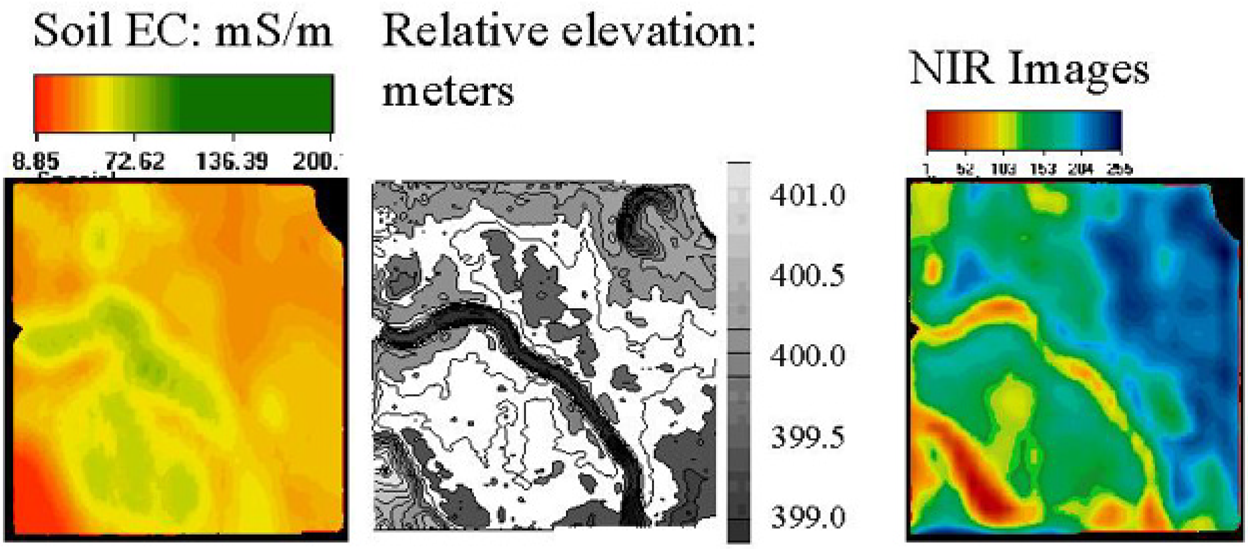

**FIGURE 7.** Soil EC, topography, and crop imagery from a 65-ha field.

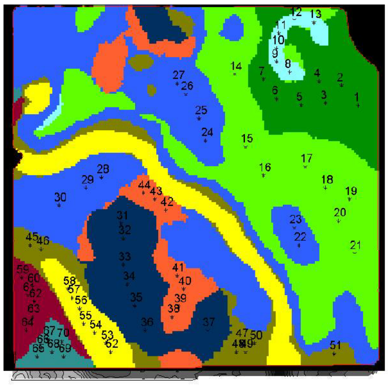

**FIGURE 8.** Zones and sampling points.

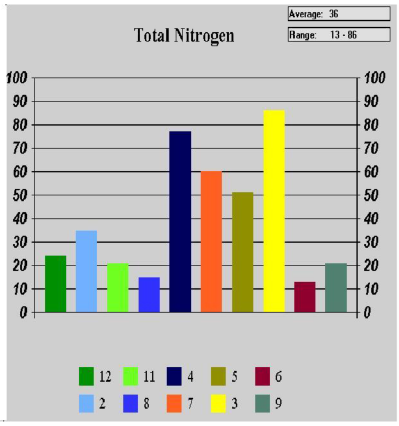

**FIGURE 9.** Nitrate N levels from each zone in Fig. 8 (kg/ha; 0- to 61-cm sampling depth).

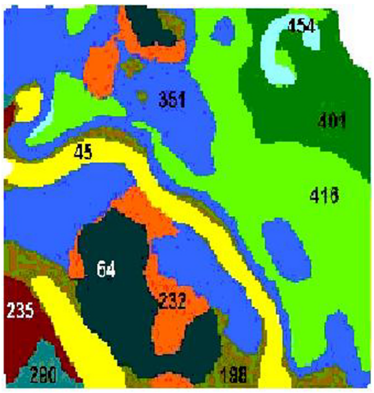

**FIGURE 10.** N recommendation (urea kg/ha).

Counties in North Dakota have shown advantages for the variable rate approach. Wheat has averaged increases of 11.3% in yield, 0.75% in protein, and $8.20/ha in net margin. Average increases on corn were 15.9% in yield, 0.70% in protein, and $16.81/ha in net margin. Soybeans had average yield increases of 9.9% and net margin gains of $10.17/ha. N usage has been affected as follows: growers who had been soil sampling previously on a whole-field basis have found their overall N rates remain about the same, with N being redistributed to zones that require differing N rates. Growers who had not been soil sampling, and who had taken a low-input approach, have found a crop response to increasing their N rates. Growers who had not been sampling previously, and who had taken an aggressive approach to fertilization, have typically been able to reduce N inputs.

## Case Study No. 2

The second case study is from central Kansas. In this 70-cm annual rainfall area, growers are making a transition from continuous wheat production to include grain sorghum, soybeans, and corn in their nonirrigated crop rotation. Soil sampling for N is typically not attempted because growers assume that the relatively mild and moist winters cause what N remains unused by previous crops to be lost over winter. Organic matter levels are in the 1 to 2% range, so only a small percentage of a crop's need for N is provided by mineralization. If N rates are to be fine-tuned in this region with available technology, it will need to be accomplished by adjusting crop yield goals.

The example field used in this case study is an 18-ha field that has soil texture and topography variability typical for fields in this region. Yield data from multiple years of various crops reveal consistently low yields in areas of this field, corresponding with eroded side slopes. High-yielding areas are the depositional areas at the bottom of these slopes. These productivity patterns are stable across a range of weather, including years with optimal rainfall (see Fig. 11).

The areas of high EC correspond to the eroded side slopes, due to the higher clay content subsoil being at or near the surface. Lower EC readings are found in the higher-yielding depositional areas, where the depth of the silt-loam topsoil is greater (see Fig. 12).

A scatter plot for EC and the 3-year normalized average yield from this field shows a statistically significant correlation at the 1% significance level (see Fig. 13). An effective method of analyzing bivariate data, such as yield and soil EC data, is boundary line analysis[14,15]. This method involves dividing EC data from a field into equal ranges, or bins. In this case, ten ranges were used. The five top-yielding points for each soil EC range were selected, which represent the highest yields in each of the ten soil EC ranges. These 50 points are plotted and a regression line is fitted to them, as in Fig. 14. This upper boundary line represents the maximum yield for each soil EC range. While there may be a number of factors causing yields to be lower than the boundary,

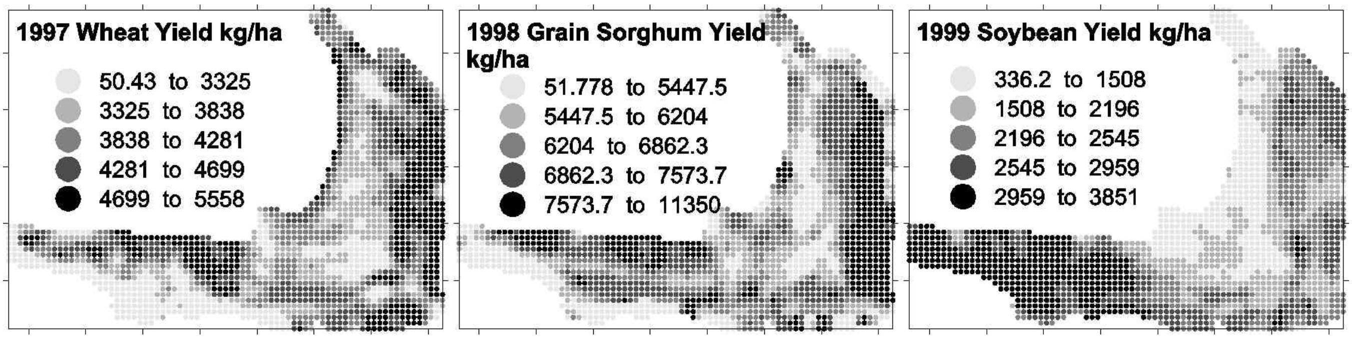

**FIGURE 11.** 1997, 1998, 1999 yields from an 18-ha Kansas field.

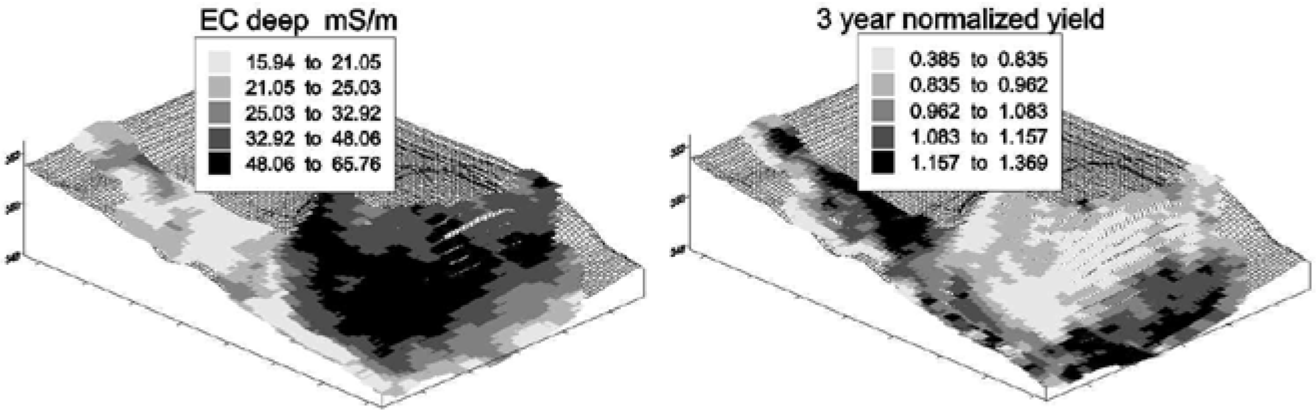

**FIGURE 12.** Soil EC and normalized 3-year yield on elevation map.

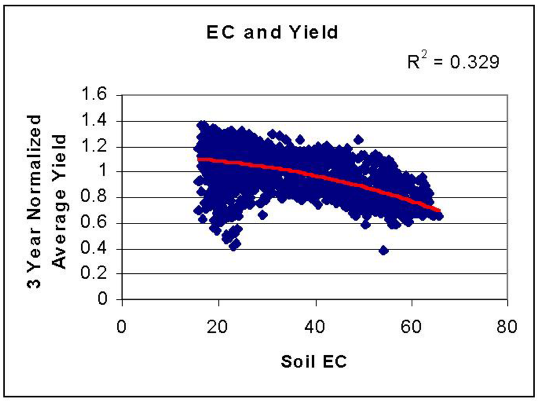

**FIGURE 13.** Scatter plot of EC and 3-year normalized average yield data.

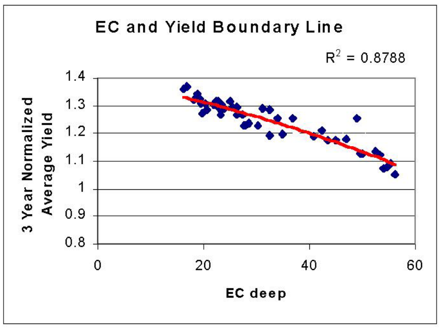

**FIGURE 14.** Boundary line plot of EC and 3-year normalized yield data.

the maximum yield for each soil EC range is delineated by the boundary line for the crop year(s) being considered. When this approach is applied to the 3 years of yield and EC data for the Kansas field in this case study, the results show a nearly linear reduction in historical productivity as soil EC increases (see Fig. 14).

This information is then used to create a yield goal map with a reduction in N for those areas that have demonstrated lowered productivity (see Fig. 15).

The analysis method described above utilizes a gridding or rasterizing of the EC and yield data in order to identify the historical maximum productivity. Other EC and yield data analysis methods are being used by consultants and growers to delineate productivity potential. The most common of these involves creating management zones based on EC. These polygons of homogeneous soil EC are queried for their average and maximum historical yields, and those results help establish a yield goal for each zone.

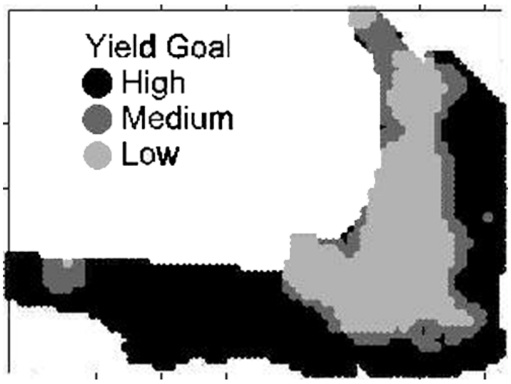

**FIGURE 15.** Yield goal map based on 3-year historical yields and soil EC data.

Before adjusting N rates based on historical yields, it is important to ensure that previous yields were not N-limited, by conducting N strip trials to evaluate the N efficiency of the various soils in the field.

## Case Study No. 3

The third case study is from the cotton-growing area of south Louisiana, in the Mississippi Delta. The traditional N practice here is for growers to use the university extension's recommendation for cotton, which is a single rate for a field based on soil type and texture, crop rotation, and cotton variety[16]. Growers and their consultants fine-tune these single rates based on their experience. Due to the nature of the alluvial soils in the Delta, there are frequently wide variations of soil texture within a field. This causes a large disparity of N availability, especially when cotton follows heavily fertilized corn in the crop rotation. Over winter, denitrification removes most of the N from the clayey soils, but silt loam and sandy loam soils often retain significantly more of the applied N into the next cropping season, in some cases enough to supply the complete N needs of the following cotton crop. On the fertile alluvial soils of the Delta, overapplication of N in cotton production often results in more adverse effects than underapplication. N applied in excess of the needs of the cotton crop results in vegetative or rank plant growth rather than increased boll production, as well as reduced fiber quality. Rank growth must be controlled by application of growth

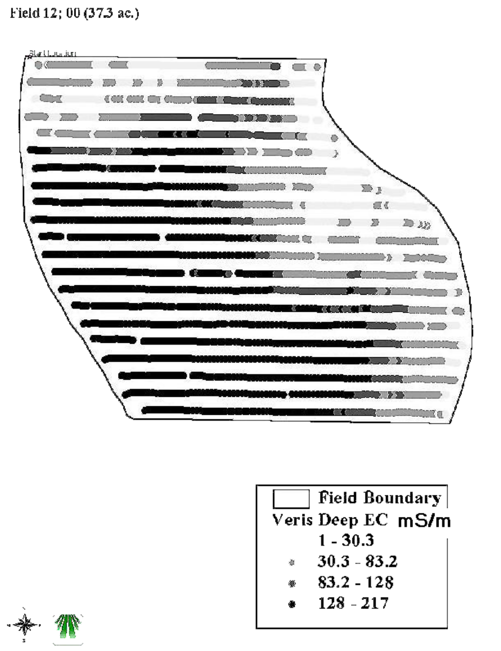

**FIGURE 16.** Soil EC data from a 15-ha Louisiana field.

regulating chemicals to reduce yield losses due to boll rot and insect damage. Increased rates of defoliant chemicals at harvest time may also be required as a result of the rank growth. The wasted N and the additional chemicals have a negative effect on the economic returns to the growers and likely the environment as well.

To improve this situation, Lambert Crop Consulting of Innis, LA is working with its clients to site-specifically apply N to cotton fields. Their approach begins with using a soil EC map to define the areas of contrasting soil texture, as in Fig. 16. Using the grower's and consultant's knowledge of the field to help interpret the soil EC data, and taking into account the crop rotation, an N recommendation is devised (see Fig. 17). Note: this process does not include soil sampling. If the grower and consultant were not familiar with the fields and areas of historical rank growth, a sampling step would likely be necessary.

The results of N applications are shown in Fig. 18. The effect of applying a single rate is evident in the 135-cm tall cotton in coarse-textured soil shown in Fig. 18A. The photographs in Fig. 18B and 18C were taken at the locations marked on the N recommendation map shown in Fig. 17. These low EC and high EC soils received the variable rate N shown in Fig.17, and cotton in both fields are 95 cm tall, the ideal height for cotton at this stage.

The results of this approach are encouraging. On the 15-ha field shown in Fig. 17 more than 10% of the field received no N and more than half of the field received less than a conventional uniform rate. Since a 30% overapplication of N to cotton has been shown to result in profit reductions of $10 to $30/ha[15]

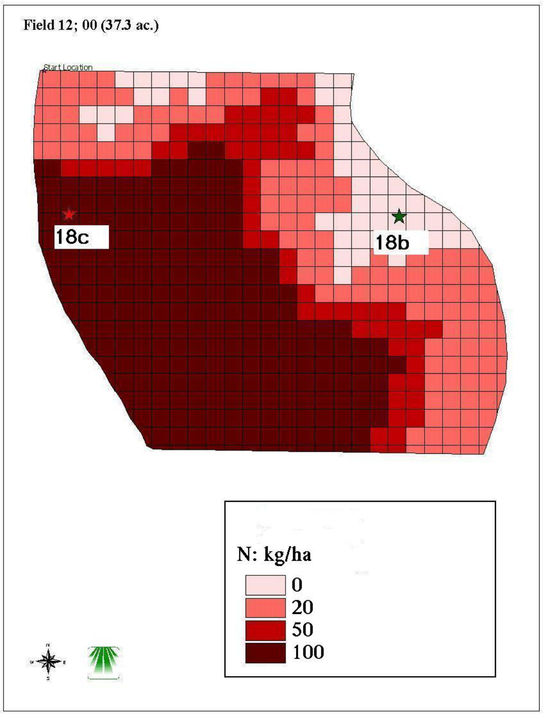

**FIGURE 17.** N recommendation map.

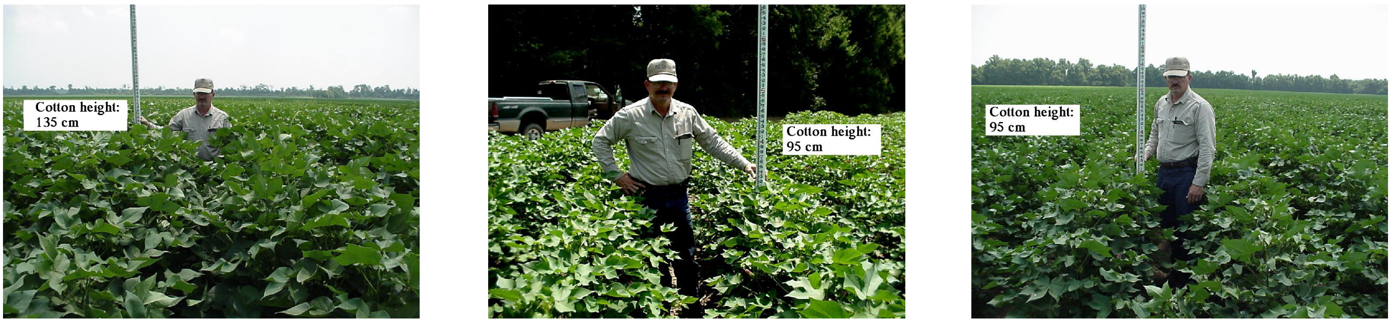

**FIGURE 18.** (A) Cotton growing in sandy soil that received a single rate of 50 kg of N/ha; (B) a sandy soil area of the adjacent field that received 0 N/ha; (C) a clayey soil area in the same field that received 100 kg of N/ha.

growers have significant financial incentives for adopting a site-specific N program. As a result, grower interest in the program is increasing, with about 1,000 ha enrolled in the system in 2001.

## SUMMARY

While research continues on the subject of proper N usage, it is feasible to fine-tune N management by applying the accepted methodologies for whole-field N rates to subfield areas, using precision agricultural technologies. Commercial input suppliers and growers have already begun the process, and as economical and environmental pressures increase, the incentive to further develop the practice will increase as well.

| | | |
|---|---|---|
| Received: | July | 18, 2001 |
| Revised: | September | 7, 2001 |
| Accepted: | September | 10, 2001 |
| Published: | October | 17, 2001 |
